# Supplementary material for: α-Clustering in atomic nuclei from first principles with statistical learning and the Hoyle state character
Source: Nat Commun. 2022 Apr 27;13:2234. doi: 10.1038/s41467-022-29582-0 (PMC9046222; doi:10.1038/s41467-022-29582-0)
Supplement: Supplementary file 1 — Supplementary Information [file 41467_2022_29582_MOESM1_ESM.pdf]

α-Clustering in Atomic Nuclei from First Principles with Statistical Learning and the Hoyle State Character: Supplementary information

T. Otsuka,[1,2,3,*] T. Abe,[2,4] T. Yoshida,[5,4] Y. Tsunoda,[4] N. Shimizu,[4]
N. Itagaki,[6] Y. Utsuno,[3,4] J. Vary,[7] P. Maris,[7] and H. Ueno[2]

[1] *Department of Physics, The University of Tokyo, 7-3-1 Hongo, Bunkyo, Tokyo 113-0033, Japan*
[2] *RIKEN Nishina Center, 2-1 Hirosawa, Wako, Saitama 351-0198, Japan*
[3] *Advanced Science Research Center, Japan Atomic Energy Agency, Tokai, Ibaraki 319-1195, Japan*
[4] *Center for Nuclear Study, The University of Tokyo, 7-3-1 Hongo, Bunkyo, Tokyo 113-0033, Japan*
[5] *Research Organization for Information Science and Technology, 2-4, Shirakata, Tokai-mura, Ibaraki-ken 319-1106, Japan*
[6] *Yukawa Institute for Theoretical Physics, Kyoto University, Kitashirakawa Oiwake-Cho, Kyoto 606-8502, Japan*
[7] *Department of Physics and Astronomy, Iowa State University, Ames, Iowa 50011, USA*

(Dated: April 8, 2022)

Supplementary Note 1. Two-Dimensional Presentation of Density Profiles

Supplementary Figure 1 displays the same panels as those in Figure 6, in the form of the two-dimensional presentation. The legend (color code) is the same as the one for Figure 6. The overlap probabilities of the regional Q-aligned states (see Figure 6 **f-i**) are also indicated. Panel **j** is added in Supplementary Figure 1 compared to Figure 6.

From this two-dimensional figure, the distance between density peaks and the angles among three density peaks can be seen more precisely than from Figure 6, although the basic features of the density distribution can be seen more clearly by the three-dimensional presentation in Figure 6.

Supplementary Note 2. Nucleon-Nucleon Interactions

JISP16 interaction[1]: The nomenclature of this NN interaction comes from the J-matrix Inverse Scattering Potential tuned up to ^{16}O (JISP16). The JISP16 interaction is a nonlocal NN potential, aiming to minimize the $3N$-force effects on *ab initio* nuclear structure calculations. No explicit three-nucleon terms are included in the JISP16 interaction, but momentum-dependent NN interaction terms produce similar effects. This potential is constructed by using a unitary transformation, the so-called phase-equivalent transformation, in order to tailor the original NN interaction, keeping two-body physical observables unchanged. Through its construction, this interaction fits not only the two-nucleon scattering data and deuteron properties, but also approximately fits the binding energies of selected nuclei up to ^{16}O.

Daejeon16 interaction[2]: The Daejeon16 NN interaction is a successor of the JISP16 NN interaction. This interaction has recently been developed and applied to *ab initio* nuclear structure calculations in light nuclei. The way to construct the Daejeon16 interaction bears similarities with JISP16, using the phase-equivalent transformation. One of the main differences between these two interactions is the initial Hamiltonian to be evolved via this transformation. The initial interaction of the Daejeon16 is taken from the similarity renormalization group (SRG)-evolved chiral effective field theory (χEFT) NN interaction up to the next-to-next-to-next leading order (N3LO), while that of the JISP16 is a much simpler one, the inverse scattering tridiagonal potential.

The chiral-effective-field-theory (χEFT) interaction[3,4] proposed by Entem and Machleidt[5] was taken with $\Lambda =$ 500 MeV, up to the N3LO in the χEFT[2].

The lattice calculation[6,7] and their extension to the reaction[8] were performed with a chiral EFT interaction up to N2LO with modifications to the lattice calculations.

Supplementary Note 3. Configuration Interaction (CI) Calculation or Shell-Model Calculation

The shell-model calculation is one of the standard methods in the nuclear many-body problem. It is similar to the Configuration Interaction (CI) calculation in other fields of science. The single-particle orbits are defined first. Protons and neutrons are put into these orbits. They are called valence protons or neutrons. Slater determinants are composed of single-particle states of these valence nucleons. We can construct the Hilbert space spanned by such Slater determinants. Each matrix element of the Hamiltonian is calculated for bra and ket vectors being Slater determinants. Once all matrix elements are calculated, the matrix is diagonalized, to solve the many-nucleon Schrödinger equation. We then obtain energy eigenvalues of this Hamiltonian as well as eigenwavefunctions, from which we can calculate various physical quantities. This is an outline of the conventional

* otsuka@phys.s.u-tokyo.ac.jp

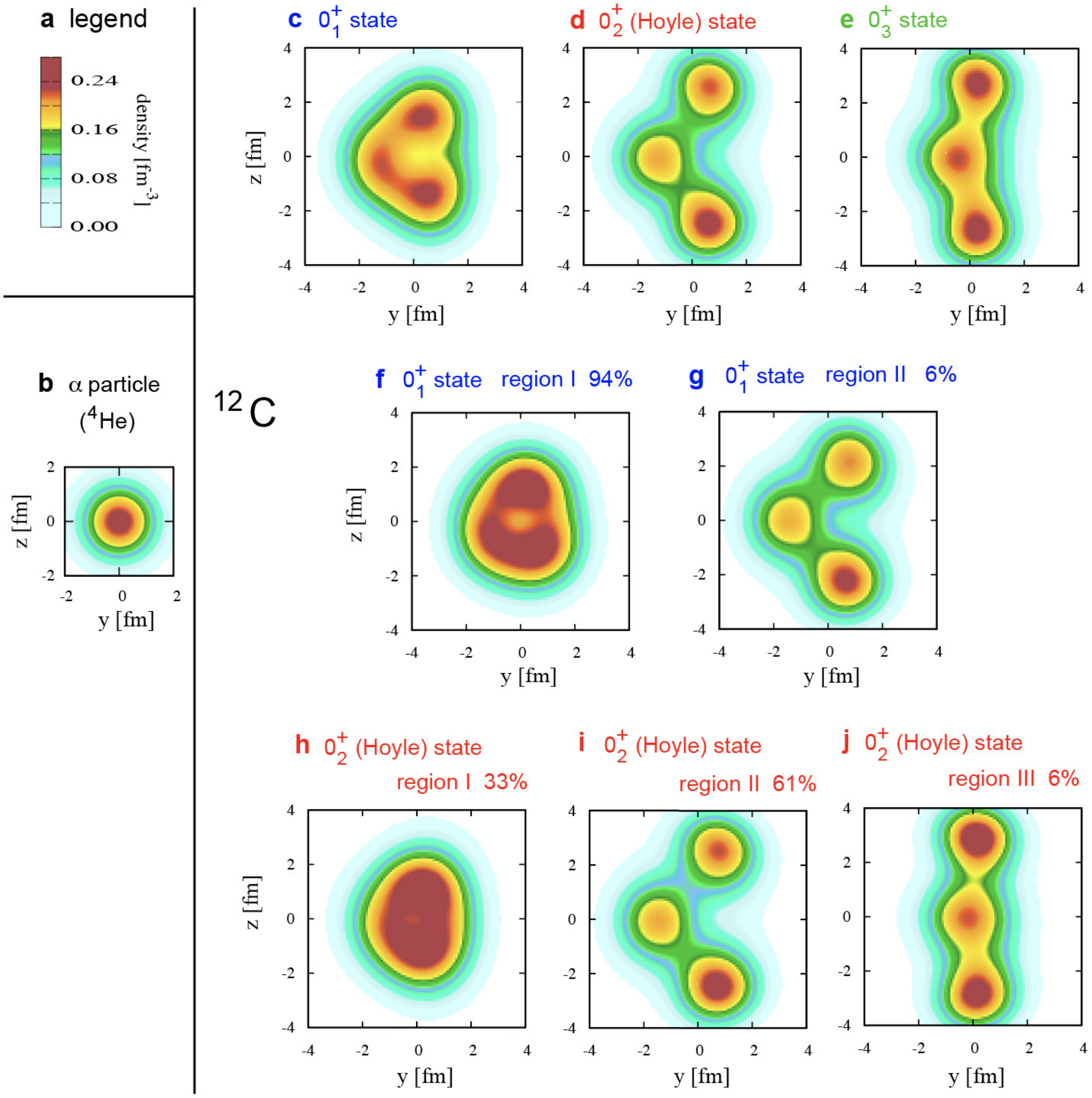

Supplementary Figure 1 | Two dimensional presentations of the density profiles on the yz plane of α and ^{12}C nuclei. All panels correspond to the panels in Figure 6. **a** Color code of the density. **b** Density of the α-particle ground state. **c-e** Density of 0^+ states of ^{12}C nucleus. **f-j** Decomposition into the regions. The probability in the indicated region is shown.

shell-model calculation. As an example of the conventional shell-model approach, the excitation level energies of ^{12}C nucleus can be calculated with some phenomenologically fitted NN interactions for small model spaces. For instance, the Cohen-Kurath interaction for the p-shell[9] can reproduce excitation energies of many states of so-called p-shell nuclei, but the Hoyle state was out of reach being more than 4 MeV away[9].

The number of Slater determinants needed in the shell-model calculation is called the shell-model dimension, and is crucial for the feasibility of actual computation. The maximum dimension, for which the conventional shell-model calculation can be performed, is about 10^{11} at present[10].

The state-of-the-art approach extending the ordinary shell model is the *ab initio* No-Core Full Configuration (NCFC). This was proposed for the calculations with *ab initio* NN interactions and has been applied to the structure of p-shell nuclei, producing salient descriptions of these nuclei (see reviews[11,12]). As an example, the ro-

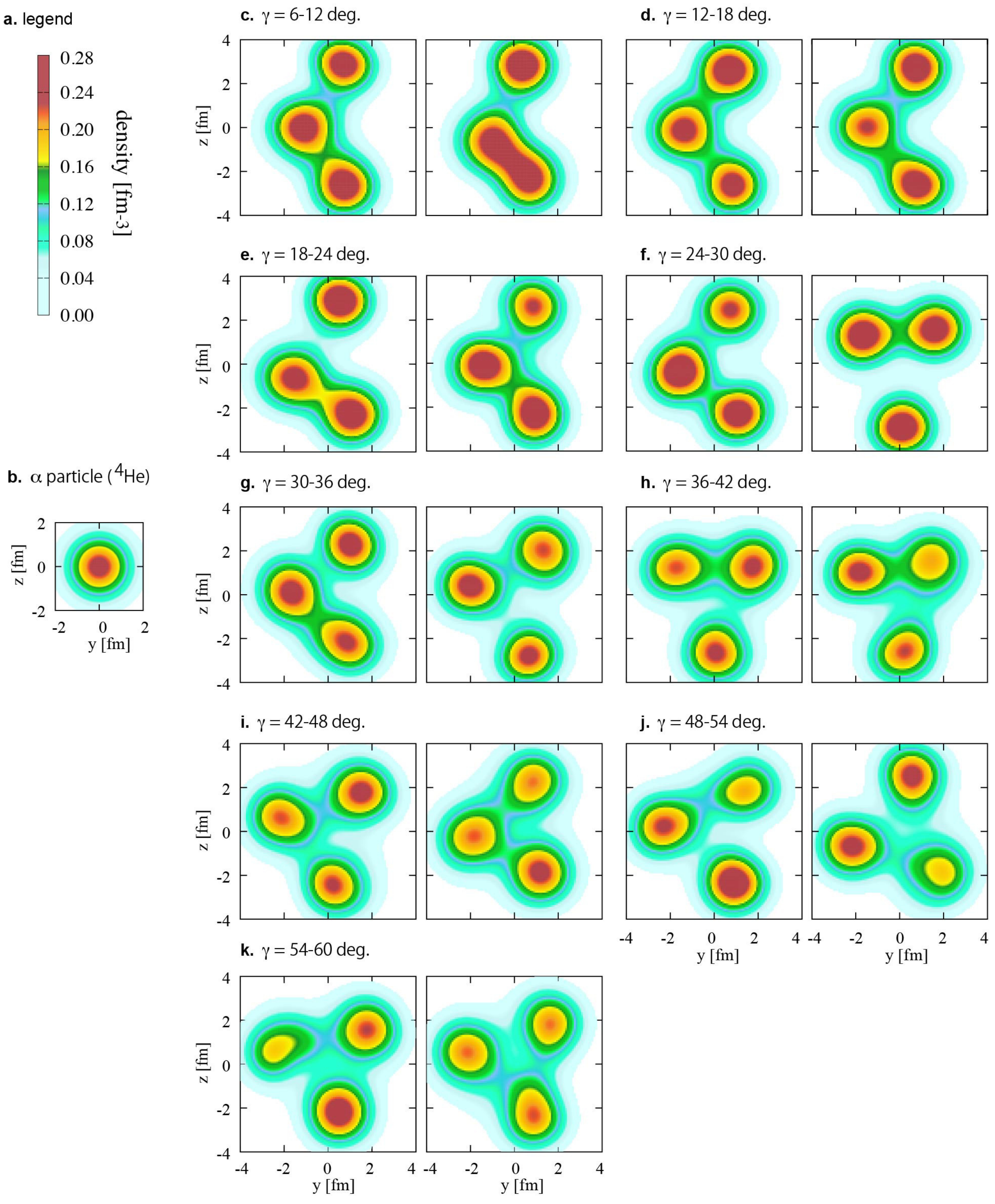

Supplementary Figure 2 | Density profiles of the MCSM basis vectors in region II of the ^{12}C nucleus. **a** Color code of the density. **b** Density of the α-particle ground state. **c-k** Density profiles on the yz plane for two basis vectors in the indicated range of γ of region II. The two basis vectors show the largest overlap probabilities with the Hoyle state.

tational bands were studied for Be isotopes, *etc.*[13,14] as mentioned in **Results**. NCFC calculations produce energy eigenvalues and $E2$ properties of ^{12}C consistent with the present work, for instance[15]. An NCFC calculation on ^{9}Be with the JISP16 interaction shows the density profile in the laboratory frame for the J^{π}=$3/2^{-}$ ground

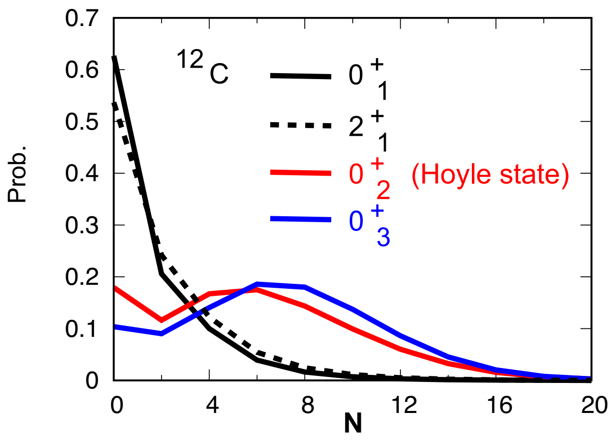

Supplementary Figure 3 | Probabilities of N$\hbar\omega$ excitations. The black, red and blue lines stand for the $0^+_{1,2,3}$ states, respectively. The dashed line represents the 2^+_1 state.

state of the ^{9}Be nucleus[16]. This density profile exhibits two peaks of the proton density apart from each other by 2 fm, whereas the peaks are about 3.5 fm away from each other in Figure 3**d**. The sharply peaked density profile in the snapshot state (or intrinsic state in literatures), like the one in Figure 3**d**, is smeared out in the density profile in the laboratory frame because of the couplings of angular momenta (see Figure 5 of ref.[16]). Although the excess neutron in ^{9}Be somewhat aligns the ground state, the laboratory-frame density profile, calculable directly from the shell-model wave function, is still far from the snapshot we find for ^{8}Be, or for ^{10}Be, that establishes clustering. Thus, this case with ^{9}Be provides another example of the crucial role of the snapshot (or intrinsic) state, as emphasized in **Results**.

In order to avoid the afore-mentioned difficulty of the shell model, the symmetry-adapted no-core shell-model (SA-NCSM) and the no-core symplectic model (NCSpM) have been proposed (see recent review[17]) and applied: the former is performed with *ab initio* interactions[18,19], while the latter is with empirical interactions[20]. The numerical calculation was made possible by truncating the many-body Hilbert space into its particular subsets with designated symmetries[21].

Another approach to overcome the difficulty of exploding shell-model dimension is the Monte Carlo Shell Model (MCSM)[22–25], which is explained in **Methods**. We note that the no-core MCSM was applied earlier to Be isotopes with more limited single-particle degrees of freedom and a different NN interaction[26].

The MCSM also has the advantage of providing a way to visualize the shape of each MCSM eigenstate through what is called the T-plot[27,28]. Because the MCSM basis vector is a deformed Slater determinant, one can calculate its intrinsic quadrupole moments, *i.e.* the quadrupole moments in the body-fixed frame. They can be expressed by two parameters β_2 and γ, as described in **Results**. The importance of each MCSM basis vector to a given eigenstate (its overlap probability in the MCSM eigenstate) is represented by the size (area) of its circular symbol in the T-plot. The T-plot is made on the PES, and intuitively exhibits the underlying physical pictures for the states of interest as demonstrated in a variety of studies, *e.g.* in Refs.[29–35].

Supplementary Note 4. Other Theoretical Approaches to the α Clustering

A shell-model approach has been reported in a different context from the viewpoint of the so-called cluster-shell competition[36,37]: the mixing of conventional shell-model states with α clustering states was discussed by using empirical NN interactions, leading to a different picture compared to the present work. No discussion was reported on triangular configurations in these works[36,37]. The α cluster structure has been considered also in other theoretical models, including the Generator Coordinator Method[38–40], the Fermion Molecular Dynamics[41], the Antisymmetrized Molecular Dynamics[42], Bose-Einstein condensate[43,44], Relativistic Mean Field[45] and the lattice-simulation[6,7] frameworks, where the Pauli principle among nucleons is activated while the wave functions are constrained dynamically. The triangular configuration appears as a key feature in the present work, similar to some other works, *e.g.*[6]. But the actual triangles seem to be rather complex and fluctuate as shown also in the present work. The relationship of our results to simple models, *e.g.* equilateral triangles[46,47], is an open and interesting question.

Supplementary Note 5. Some Observables of ^{12}C

The calculated value of $B(E2; 0^+_2 \to 2^+_1)$ of ^{12}C is somewhat smaller than the quoted experimental value. The experimental value may be changed to a larger value (personal communications from Kibédi, T., Stuchbery, A. E. and Gorgön, A. based on the data[48]). The present calculated value is larger than some other theoretical values (see, for instance, a review[44]), but turns out not to be large enough. If more single-particle orbits are included as stated above, the nucleus may be somewhat more deformed or may have somewhat more developed clustering, and thereby the present calculated value may increase. This point motivates larger calculations in the future. In such future calculations, one expects the excitation energy of the 0^+_2 (Hoyle) state to further decrease towards the experimental one.

Likewise, the radius and $E0$ transition of the Hoyle state can be improved if more single-particle orbits are

included. However, the ground state properties will likely be less influenced, as expected from Supplementary Figure 3. The transition densities of elastic and inelastic scatterings are of interest, and will be analyzed including the finite-size effects of proton and neutron.

Supplementary References

1. Shirokov, A. M., Vary, J. P., Mazur, A. I. and Weber, T. A., Realistic nuclear Hamiltonian: Ab exitu approach. Phys. Lett. B **644**, 33 (2007).
2. Shirokov, A. M., Shin, I. J., Kim, Y., Sosonkina, M., Maris P. and Vary, J. P., N3LO NN interaction adjusted to light nuclei in *ab exitu* approach. Phys. Lett. B **761**, 87 (2016).
3. Machleidt, R. & Entem, D. R., Chiral effective field theory and nuclear forces, Phys. Rep. **503**, 1 (2011).
4. Epelbaum, E., Hammer, H.-W., and Meißner, Ulf-G., Modern theory of nuclear forces, Rev. Mod. Phys. **81**, 1773 (2009).
5. Entem, D. R. and Machleidt, R., Accurate charge-dependent nucleon-nucleon potential at fourth order of chiral perturbation theory, Phys. Rev. C **68**, 041001 (2003).
6. Epelbaum, E., Krebs, H., Lähde, T. A., Lee, D. and Meißner, U.-G., Structure and Rotations of the Hoyle State, Phys. Rev. Lett. **109**, 252501 (2012).
7. Epelbaum,E., Krebs, H., Lee, D. and Meißner, U.-G., *Ab Initio* Calculation of the Hoyle State, Phys. Rev. Lett. **106**, 192501 (2011).
8. Elhatisari, S., *et al.*, *Ab initio* alpha–alpha scattering, Nature. **528**, 111 (2015).
9. Cohen, S. & Kurath, D., Effective Interactions for the 1p shell, Nucl. Phys. **73**, 1 (1965).
10. Shimizu, N., Mizusaki, T., Utsuno, Y. & Tsunoda, Y., Thick-Restart Block Lanczos Method for Large-Scale Shell-Model Calculations, Comp. Phys. Comm. **244**, 372 (2019).
11. Maris P. and Vary, J. P., *ab initio* NUCLEAR STRUCTURE CALCULATIONS OF p-SHELL NUCLEI WITH JISP16, Int. J. Mod. Phys. E **22**, 1330016 (2013).
12. Barrett, B. R., Navratil, P., & Vary, J. P., Ab initio no core shell model, Prog. Part. Nucl. Phys. **69**, 131 (2013).
13. Maris, P., Caprio, M. A. & Vary, J. P., Emergence of rotational bands in *ab initio* no-core configuration interaction calculations of the Be isotopes, Phys. Rev. C **91**, 014310 (2015).
14. Caprio, M. A., Maris P. and Vary, J. P., Emergence of rotational bands in ab initio no-core configuration interaction calculations of light nuclei, Phys. Lett. B **719**, 179 (2013).
15. Maris, P., Vary,, J. P., Calci, A., Langhammer, J., Binder, S., and Roth, R., ^{12}C properties with evolved chiral three-nucleon interactions, Phys. Rev. C **90**, 014314 (2014).
16. Maris, P., Ab Initio Nuclear Structure Calculations of Light Nuclei, J. Phys.: Conf. Ser. **402**, 012031 (2012).
17. Launey, K. D., Dytrych, T., Sargsyan, G. H., Baker, R. B., & Draayer, J. P., Emergent symplectic symmetry in atomic nuclei, Eur. Phys. J. Special Topics **229**, 2429 (2020).
18. Dreyfuss, A. C., Launey, K. D., Dytrych, T., Draayer, J. P., & Bahri, C., Hoyle state and rotational features in Carbon-12 within a no-core shell-model framework, Phys. Lett. B **727**, 511 (2013).
19. Dytrych, T., *et al.*, Collective Modes in Light Nuclei from First Principles, Phys. Rev. Lett. **111**, 252501 (2013).
20. Dreyfuss, A. C., *et al.*, Understanding emergent collectivity and clustering in nuclei from a symmetry-based no-core shell-model perspective, Phys. Rev. C **95**, 044312 (2017).
21. Dytrych, T., Sviratcheva, K. D., Draayer, J. P., Bahri, C., & Vary, J. P., *ab initio* symplectic no-core shell model, J. Phys. G, **35**, 123101 (2008).
22. Honma, M., Mizusaki, T., & Otsuka, T., Diagonalization of Hamiltonians for Many-Body Systems by Auxiliary Field Quantum Monte Carlo Technique, Phys. Rev. Lett. **75**, 1284 (1995).
23. Otsuka, T., Mizusaki, T., & Honma, M., Structure of the N=Z=28 Closed Shell Studied by Monte Carlo Shell Model Calculation, Phys. Rev. Lett. **81**, 1588 (1998).
24. Otsuka, T., Honma, M., Mizusaki, T., Shimizu, N. & Utsuno, Y., Monte Carlo Shell Model for Atomic Nuclei, Prog. Part. Nucl. Phys. **47**, 319-400 (2001).
25. Shimizu, N. *et al.*, New-generation Monte Carlo shell model for the K computer era, Prog. Theor. Exp. Phys. **2012**, 01A205 (2012).
26. Liu, L., Otsuka, T., Shimizu, N., Utsuno, Y., and Roth, R., No-core Monte Carlo shell-model calculation for ^{10}Be and ^{12}Be low-lying spectra, Phys. Rev. C **86**, 014302 (2012).
27. Tsunoda, Y., Otsuka, T., Shimizu, N., Honma, M. & Utsuno, Y. Novel shape evolution in exotic Ni isotopes and configuration-dependent shell structure, Phys. Rev. C **89**, 031301(R) (2014).
28. Otsuka, T. and Tsunoda, Y., The role of shell evolution in shape coexistence, J. Phys. G, **43**, 024009 (2016).
29. Ichikawa, Y. *et al.*, Interplay between nuclear shell evolution and shape deformation revealed by the magnetic moment of ^{75}Cu, Nature Physics **15**, 321 (2019).
30. Chiara, C. J., *et al.*., Identification of deformed intruder states in semi-magic ^{70}Ni, Phys. Rev. C **91**, 044309 (2015).
31. Togashi, T., Tsunoda, Y., Otsuka, T., & Shimizu, N., Quantum Phase Transition in the Shape of Zr isotopes, Phys. Rev. Lett. **117**, 172502 (2016).
32. Leoni, S., *et al.*., Multifaceted Quadruplet of Low-Lying Spin-Zero States in ^{66}Ni: Emergence of Shape Isomerism in Light Nuclei, Phys. Rev. Lett. **118**, 162502 (2017).
33. Marsh, B. A. *et al.*, Characterization of the shape-staggering effect in mercury nuclei, Nature Physics **14**, 1163 (2018).
34. Togashi, T., Tsunoda, Y., Otsuka, T., Shimizu, N., & Honma, M., Novel Shape Evolution in Sn Isotopes from Magic Numbers 50 to 82, Phys. Rev. Lett. **121**, 062501 (2018).
35. Otsuka, T., Tsunoda, Y., Abe, T., Shimizu, N. & Van Duppen, P., Underlying Structure of Collective Bands and Self-Organization in Quantum Systems, Phys. Rev. Lett. **123**, 222502 (2019).
36. Itagaki, N., Aoyama, S., Okabe, S. and Ikeda, K., Cluster-shell competition in light nuclei, Phys. Rev. C **70**, 054307 (2004).
37. Itagaki, N., Aoyama, S., Okabe, S. and Ikeda, K., Novel and simple description for a smooth transition from α-cluster wave functions to jj-coupling shell model wave functions, Phys. Rev. C **87**, 054334 (2013).

38. Uegaki, E., Okabe, S., Abe, Y. & Tanaka, H., Structure of the Excited States in ^{12}C. I. Prog. Thoer. Phys., **57**, 1262 (1977).
39. Kamimura, M., Transition densities between the 0_1^+, 2_1^+, 4_1^+, 0_2^+, 2_2^+, 1_1^- and 3_1^- states in ^{12}C derived from the three-alpha resonating-group wave functions. Nucl. Phys. A **351**, 456 (1981).
40. Ikeda, K., Horiuchi, H. and Saito, S., Introduction to Comprehensive Nuclear Structure Study Based on Cluster Correlations and Molecular Viewpoint. Prog. Thoer. Phys. Suppl., **No. 68**, 1 (1980).
41. Chernykh, M., Feldmeier, H., Neff, T., Von Neumann-Cosel, P., Richter, A. Structure of the Hoyle State in C12, Phys. Rev. Lett. **98**, 032501 (2007).
42. Kanada-En'yo, Y., The Structure of Ground and Excited States of ^{12}C, Prog. Thoer. Phys., **117**, 655 (2007).
43. Tohsaki, A., Horiuchi, H., Schuck, P. & Röpke, G. Alpha cluster condensation in ^{12}C and ^{16}O. Phys. Rev. Lett. **87**, 192501 (2001).
44. Funaki, Y., Horiuchi, H., and Tohsaki, T., Cluster models from RGM to alpha condensation and beyond. Prog. Part. Nucl. Phys., **82**, 78 (2015).
45. Zhao, P. W., Itagaki, N. and Meng, J., Rod-shaped Nuclei at Extreme Spin and Isospin, Phys. Rev. Lett. **115**, 022501 (2015).
46. Bijker, R., and Iachello, F., The Algebraic Cluster Model: Three-Body Clusters. Ann. Phys. **298**, 334 (2002).
47. Marín-Lámbarri, D. J., Bijker, R., Freer, M. , Gai, M., Kokalova, Tz., Parker, D. J. & Wheldon, C., Evidence for Triangular D_{3h} Symmetry in ^{12}C. Phys. Rev. Lett. **107**, 112501 (2011).
48. Kibédi, T. *et al.*, Radiative Width of the Hoyle State from γ-Ray Spectroscopy, Phys. Rev. Lett. **125**, 182701 (2020).
